# A Flexible TENG Based on Micro-Structure Film for Speed Skating Techniques Monitoring and Biomechanical Energy Harvesting

**DOI:** 10.3390/nano12091576

**Published:** 2022-05-06

**Authors:** Zhuo Lu, Changjun Jia, Xu Yang, Yongsheng Zhu, Fengxin Sun, Tianming Zhao, Shouwei Zhang, Yupeng Mao

**Affiliations:** 1 School of Physical Education, Northeast Normal University, Changchun 130024, China; luz560@nenu.edu.cn; 2 Physical Education Department, Northeastern University, Shenyang 110819, China; 2001276@stu.neu.edu.cn (Y.Z.); 2171435@stu.neu.edu.cn (F.S.); 3 Changchun Polytechnic Tourism School, Changchun 130022, China; yangxu0316@126.com; 4 College of Sciences, Northeastern University, Shenyang 110819, China; zhaotm@stumail.neu.edu.cn

**Keywords:** self-powered, wearable flexible sensor, energy harvesting, human motion monitoring, triboelectric nanogenerator

## Abstract

Wearable motion-monitoring systems have been widely used in recent years. However, the battery energy storage problem of traditional wearable devices limits the development of human sports training applications. In this paper, a self-powered and portable micro-structure triboelectric nanogenerator (MS-TENG) has been made. It consists of micro-structure polydimethylsiloxane (PDMS) film, fluorinated ethylene propylene (FEP) film, and lithium chloride polyacrylamide (LiCl-PAAM) hydrogel. Through the micro-structure, the voltage of the MS-TENG can be improved by 7 times. The MS-TENG provides outstanding sensing properties: maximum output voltage of 74 V, angular sensitivity of 1.016 V/degree, high signal-to-noise ratio, and excellent long-term service stability. We used it to monitor the running skills of speed skaters. It can also store the biomechanical energy which is generated in the process of speed skating through capacitors. It demonstrates capability of sensor to power electronic calculator and electronic watch. In addition, as a flexible electrode hydrogel, it can readily stretch over 1300%, which can help improve the service life and work stability of MS-TENG. Therefore, MS-TENG has great application potential in human sports training monitoring and big data analysis.

## 1. Introduction

In the Beijing Winter Olympic Games, a total of 14 gold medals were won in speed skating, including 10 Olympic records and 1 world record. The good results of athletes are inseparable from scientific training. Among this, the data support provided by sports training monitoring for speed skating training and competition is the key link. The speed skating competition is fierce. Speed skaters wear 2 mm-wide skates to complete high-speed skating on the ice. High-quality athletes’ technical motions are the fundamental guarantee of this high-speed movement. The athletes’ physical agility is the main influencing factor of their competitive ability and excellent performance. Moreover, the physical agility decline caused by technical instability exists in most athletes’ training and competition [1,2,3,4,5,6]. High speed cameras and inertial sensors have been used to monitor the changes in athletes’ real-time technical motions during taxiing [7,8,9,10]. However, the accuracy of motion monitoring is limited by the large space demand, complex circuit, and large battery volume of cameras, inertial sensors, and portable sensors. Therefore, it is an urgent problem to develop a portable, economical, self-powered, and real-time motion monitoring sensor to assist the development of speed skating.

In recent years, the Zhong Lin Wang team invented the triboelectric nanogenerator (TENG) based on Maxwell displacement current theory [11,12,13], which has been widely discussed and developed rapidly. This technology has a great application potential in the fields of blue energy, self-powered systems and portable sensors [14,15,16,17,18,19]. TENG mainly consists of two different materials [20,21,22,23]. It can convert low-frequency mechanical energy from surroundings into electrical energy, such as human motion mechanical energy [24,25,26,27,28,29]. Due to the electrical signal being closely related to the surroundings, TENG seems to be an ideal candidate for motion monitoring. Unfortunately, common TENG with a metal electrode can be easily destroyed and uncomfortable to wear, so it cannot be further applied to biological systems [30,31]. Chen et al. propose a kind of hydrogel with high conductivity, transports and flexibility [32,33]. Combing with conductive hydrogel and surface modification with micro-structure to fabricate TENG, proposed by Zhao et al. [34,35,36], the sensitivity and response of the self-powered sensor would be dramatically enhanced.

**Figure 1:**
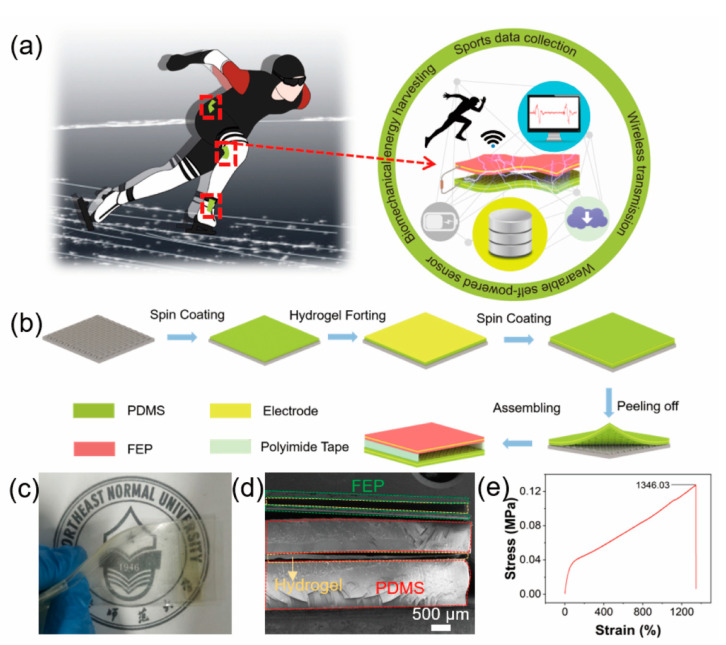
(**a**) The application scenarios of MS-TENG; (**b**) the fabricating process of the MS-TENG; (**c**) the optical image of MS-TENG at bending state; (**d**) the SEM image of the MS-PDMS; (**e**) tensile strength testing of hydrogel.

In this work, we develop a micro-structure triboelectric nanogenerator (MS-TENG). It consists of micro-structure polydimethylsiloxane (PDMS) film, fluorinated ethylene propylene (FEP) film, and lithium chloride polyacrylamide (LiCl-PAAM) hydrogel (Figure 1). Through introduction of micro-structure on dielectric surface, the output voltage of the MS-TENG can be improved by 7 times. In our experiment, MS-TENG can be attached to the athlete’s body surface easily and it can collect the technical motion information accurately (movement structure, bending angle and frequency). The triboelectric signal can not only be used as biosensor signal, but also can power microelectronics. In addition, by replacing metal electrodes with hydrogel, the response, stability, lifetime and comfort level have been improved. Therefore, MS-TENG can be applied to sports training monitoring and big data analysis of speed skating or other sports. As a new generation of motion-monitoring equipment, it has great application potential.

## 2. Materials and Methods

### 2.1. Materials

Fluorinated ethylene propylene and Polyimide tape were purchased from Zeyou plastic Co., Ltd. (Suzhou, China). DOW CORNING Sylgard 184 was purchased from Xinheng Trading Co., Ltd. (Tianjin, China). N,N-dimethylformamide (DMF), Acrylamide (AM), Lithium chloride (LiCl), N,N’-methylene diacrylamide (MBA), Ammonium persulphate (APS), and N,N,N’,N’-tetramethylethylenediamine (TMEDA) were purchased from Jintong letai chemical industry products Co., Ltd. (Beijing, China).

### 2.2. Methods

*Synthesis of lithium chloride polyacrylamide hydrogel pre-solution:* AM was used as the monomer, MBA was used as the crosslinking agent, and APS was used as the initiator. The whole reaction was processed under room temperature. The specific steps were as follows: AM powder and LiCl particles were dissolved in 50 mL deionized water at a speed of 500 rpm, wherein the concentrations of AM and LiCl were 3 mol/L and 5 mol/L, respectively. After continuous magnetic stirring for 10 min, MBA and APS were added to the solution, and the molar ratios of MBA and APS to AM monomer were 0.02 and 0.03 mol% respectively. Then, the particles were stirred until they were dissolved completely, and then it was kept for 1 h to obtain the pre-solution.

*Preparation of micro-structure PDMS triboelectric layer:* The PDMS mixture of base and crosslinker (the weight ratio of base to cross linker was 10:1) was stirred for at least 20 min and degassed in vacuum for 10 min to remove air bubbles at room temperature. PDMS mixture was spin-coated (900 rpm, 20 s) on a silicon mold with microstructure, and cured at 80 °C for 1 h. A few drops of TMEDA was added in the uniform hydrogel solution which is used as an accelerator. Subsequently, the hydrogel solution was spin-coated on PDMS. After the hydrogel was solidified, the PDMS mixture was spin-coated on the above hydrogel membrane again. After the PDMS was solidified, PDMS films with micro-structure were obtained by peeling off the sandwich PDMS from the Si mold surface carefully.

*Manufacture of triboelectric nanogenerator:* The FEP triboelectric layer with sandwich structure was composed of FEP film and LiCl-PAAM hydrogel. Finally, the double-electrode TENG with microstructure (MS-TENG) consisted of PDMS triboelectric layer, FEP triboelectric layer, and polyimide (50 µm). Polyimide was used as spacer layer, which provided space for two triboelectric layers.

### 2.3. Characterization and Measurement

The MS-TENG was fixed on the stepping motor to simulate joint movement. The different amplitudes and frequencies were used to hit sensors repeatedly and periodically and triboelectric single was generated by the MS-TNEG. Signals were collected by oscilloscopes (sto 1102 c, Shenzhen, China). The morphology and structure of the sensor were carried out by an optical microscope (Sunshine Instrument Co., Ltd., SDPTOP-CX 40m, Ningbo, China).

## 3. Results and Discussion

To achieve accurate, reliable, and convenient assessment of the technical movements of speed skaters, conformal and real-time measurement of athlete’s joint and articular chains is necessary. We proposed a self-powered and flexible sensor (MS-TENG) which consists of PDMS elastomer, FEP film, and ionic conductive hydrogel. According to MS-TENG output signals, a coach can adjust a skater’s technical movements and develop a suitable plan for an athlete, so that they can scientifically and systematically enhance athlete’s performance. As shown in Figure 1a, MS-TENG can attach to the joints of the skater flexibly. Based on the triboelectric effect, the output signals of MS-TENG are sensing signals. It can collect the information of athletes’ joint bending angle, movement frequency and movement structure, and it provides the basis for big data analysis. We have made a comparison between the existing articles in the field of manufacturing sensors that we have referred to in this research. The results of this comparison are shown in Table S1 [37,38,39,40,41,42,43,44]. Compared with other works, the MS-TENG has the advantages of self-powered, soft, and high-outputting properties. Hydrogel has been used as flexible electrode, which improves the service life and working stability of MS-TENG. The application value of the sensor has been verified. The manufacture process of MS-TENG is shown in Figure 1b. In brief, the PDMS mixture is spin-coated on a silicon mold. After curing, the complementary structure of the epidermis pattern is uniformly transferred from the silicon mold to the PDMS. Later, the hydrogel pre-solution with TMEDA is spin-coated on the bottom PDMS layer, and then the PDMS mixture is spin-coated above the hydrogel again. After curing, a microstructure PDMS triboelectric layer can be obtained. Finally, the PDMS triboelectric layer and FEP triboelectric layer are assembled together by polyimide tape. Figure 1c is an optical image of the MS-TENG at bending state. It shows the flexible, soft, and thin characteristics of MS-TENG. Figure 1d shows the cross-sectional scanning electron microscope (SEM) image of MS-TENG which clearly shows the structure of MS-TENG. Figure S1 shows the SEM images of PDMS, FEP, and hydrogel, respectively. The microstructure of PDMS surface is shown in Figure S1a. Since MS-PDMS has a large effective contact area and higher surface energy. It leads to more charge accumulation on the contact surface, higher potential, and better electrical performance. Figure S2 shows the Fourier-transform infrared (FTIR) spectrum of the FEP, PDMS, and hydrogel. Then we investigated the mechanical properties of hydrogel, which is an important parameter for practical applications. The tensile strength measurement process of hydrogel is shown in Figure S3. As shown in Figure 1e, hydrogel can stretch over 1300%, which compared with traditional metal electrode, hydrogel electrode has excellent flexibility [45,46]. In addition, this work expands the application of hydrogels in other fields [47,48,49].

**Figure 2:**
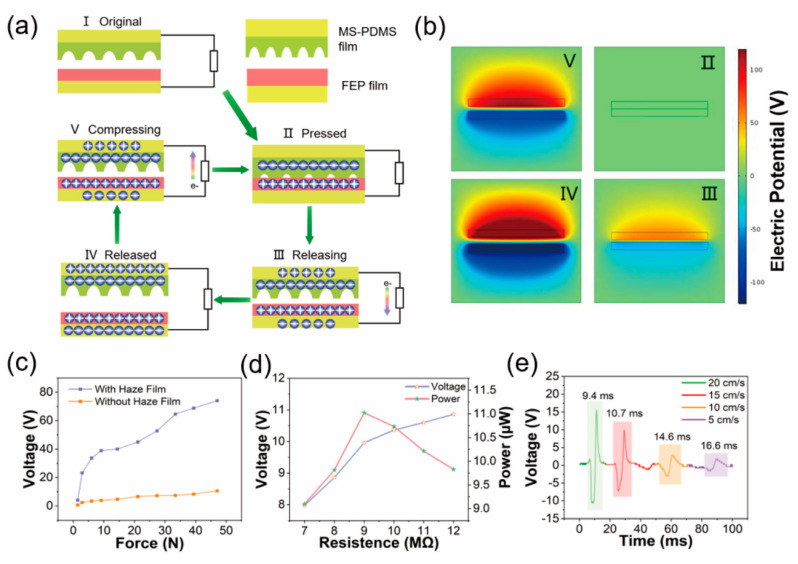
(**a**) Working principle of the MS-TENG; (**b**) FEA simulation of the MS-TENG electric potential distribution; (**c**) peak voltage of MS-TENG and without microstructure TENG under variable applied force from 0 to 50 N; (**d**) the output performance of MS-TENG under different load resistances; (**e**) the voltage wave of MS-TENG under speed of 5, 10, 15, and 20 cm/s.

The working mechanism of the MS-TENG is schematically illustrated in Figure 2a. In the original state (Figure 2a(I)), charge transfer does not take place before the triboelectric materials contacts. When a pressure force is applied to MS-TENG (Figure 2a(II)) charge transfer takes places at the interface between PDMS and FEP, due to the differences of the electronegativity [50]. Since the surface electron affinity of PDMS is higher than FEP, electrons transfer from the FEP surface to PDMS surface, leaving equal positive triboelectric charges on the FEP surface. When a pressure force disappears, PDMS and PEF begin to separate and the electrons transfer from the top electrode to the bottom electrode via external circuit due to the electrostatic force (Figure 2a(III)). As shown in Figure 2a(IV), the MS-TENG reaches the equilibrium state, and electrons do not transfer from the top electrode to the bottom electrode anymore. Finally, when the pressure force appears again, the electrons transfer from bottom electrode to top electrode via external circuit due to the electrostatic force (Figure 2a(V)), outputting a reversing electrical signal. Therefore, the AC electricity can be continuously generated by periodical contact-separation between PDMS film and FEP film. In order to understand the working mechanism of the MS-TENG, the visualized simulation via COMSOL software is shown in Figure 2b, and the corresponding simulated output electric potential is depicted by color variation. We measure the peak voltage of MS-TENG and without microstructure TENG under variable applied force from 0 to 50 N (as shown in Figure 2c). It shows that the two peak voltages increase with the increase of force, but the MS-TENG is more sensitive to response of force. The output performance of MS-TENG under different load resistances is shown in Figure 2d. The output voltage increases with the load resistance increasing. Instantaneous electric power is the 11 μW at 9 MΩ. Meanwhile, we tested the resistance of the hydrogel. When the hydrogel is stretched from 1 cm to 15 cm, the resistance increases rapidly and then stabilizes gradually, the maximum resistance reaches 564.2 kΩ (Figure S4). Even if the resistance of hydrogel changes with the stretching, the inherent resistance of MS-TENG is much larger than that of the hydrogel. Therefore, the change of hydrogel resistance does not affect the output voltage. In order to explore the characteristics of the TENG generator, the energy conversion efficiency of the MS-TENG is investigated. The efficiency of the MS-TENG is defined as the ratio between the input mechanical energy and the generated electrical energy delivered to the load. The formula of energy conversion efficiency are as follows:

(1) $$\eta =\frac{E_{\mathrm{ele}}}{W_{total}}=\frac{\int I^{2}Rdt}{W_{G}}$$

where *E*_ele_ and *W_total_* stand for the electric energy and the total work done by the ambient, *W_G_* represents the work done by gravity. As shown in Figure S5, *E*_ele_ shows an energy output that is measured under the best matched load (9 MΩ). According to calculation, the energy conversion efficiency of TENG is 0.08%. Figure 2e shows the voltage wave of the MS-TENG, when the pressing/releasing speed is from 5 to 20 cm/s. It can be observed that the peak voltage decreases with the pressing/releasing speed decreasing also, and the pulse width increases gradually with the pressing/releasing speed decreasing.

**Figure 3:**
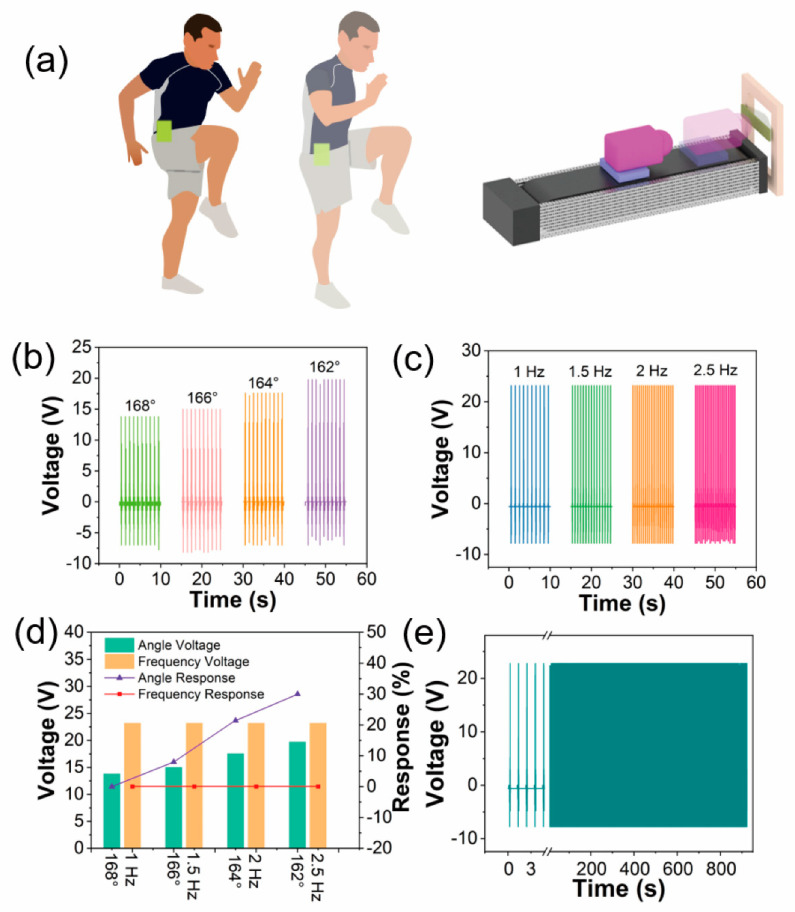
(**a**) MS-TENG monitors the body joint motion system; (**b**) outputting triboelectric voltage of MS-TENG at different bend angles; (**c**) outputting triboelectric voltage of MS-TENG at different frequencies; (**d**) response of output triboelectric voltage of MS-TENG at different bending angles and frequencies; (**e**) durability property of MS-TENG.

The physiological structure of the human body determines the working mode of the upper and lower limbs. Combined with torso coordination and cooperation, various forms of human movement are formed. With the body movement forms changing, many movements are formed such as push, pull, stretch, swing, among others. Further, many motion of human can drive TENG to work. Before practical application, it is necessary to study the effects of different mechanical stimuli on the output of MS-TENG to prove its practicability. Figure 3a is a system of MS-TENG monitoring body joint movements. We manufactured a MS-TENG which size is 8 × 3 cm^2^. The stepping motor with programmable system and slide rail simulates joint movement to apply different deformations to the sensor. All the measurements are carried out at room temperature (22 °C) and 25% relative humidity. The output triboelectric voltage of the MS-TENG at the same frequency (1 Hz) and different bending angles (as shown in Figure 3b). When the angles are 168, 166, 164, and 162°, the output triboelectric voltage is 13.8, 15, 17.56, and 19.27 V, respectively, and the output voltage increases with the bending angle increasing. In order to show the sensitivity of MS-TENG, the linear relationship between bending angles and output voltages is shown in Figure S6. The red line is a linear fit. The linear fitting of Formula (2) is as follows:

(2) y=184.16−1.016x

where *y* represents the triboelectric voltage (V) and *x* represents the bending angle (degree). The linearity is up to 0.99. Figure 3c shows the relationship between output triboelectric voltage and frequency. When the bending angle is 160°, the frequencies are 1, 1.5, 2, and 2.5 Hz, and the output triboelectric voltages are 1.85, 1.88, 1.86, and 1.92 V respectively. Figure 3d shows the response of MS-TENG at different bending angles and frequencies. The response of MS-TENG can be calculated from the following equation:

(3) $$\%=\left|\frac{V_{0}-V_{i}}{V_{i}}\right|\times 100\%,$$

where *V*_0_ and *V_i_* are the outputting voltage of 168° (first data) and other voltages. The response of MS-TENG is 0, 8, 21.4, and 30% when it is in different bending angles, and when the frequency is 1, 1.5, 2, and 2.5 Hz, the response of MS-TENG is 0, 0, 0, and 0%. These data indicate that MS-TENG can monitor the joint change of angle and frequency accurately, and these data are used be big data analysis to enhance athlete’s sports technology. The durability of MS-TENG is shown in Figure 3e. After many tests, the output is almost constant (~22 V). The excellent durability and high output power of the MS-TENG shows the potential of practical application in the future.

**Figure 4:**
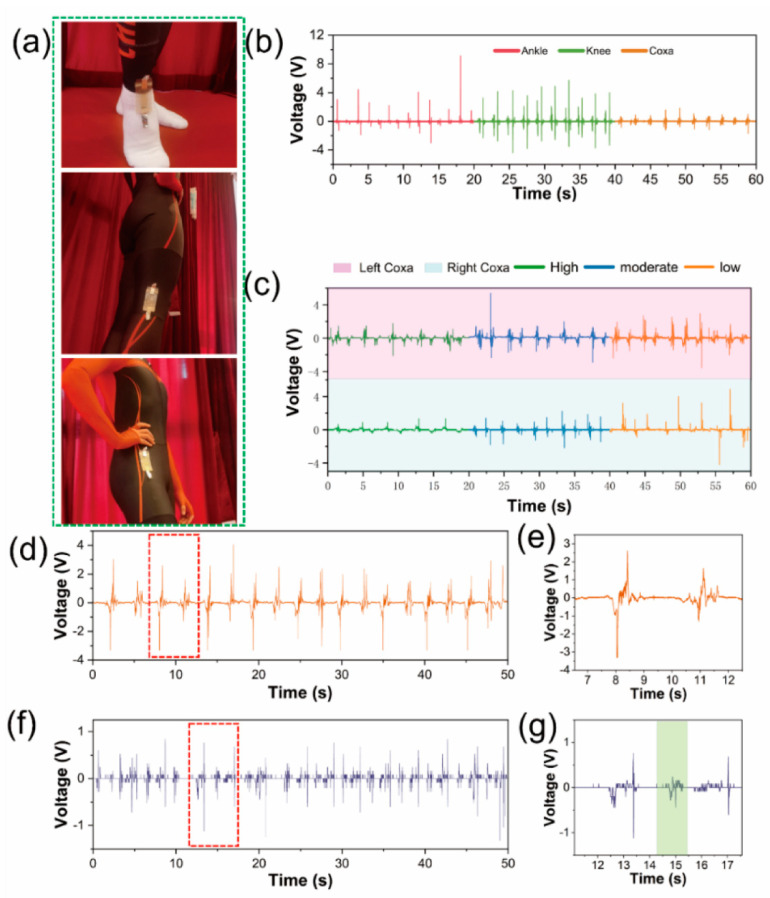
(**a**) Images of the MS-TENG attached to the ankle, knee, and coxa; (**b**) the output triboelectric voltage of MS-TENG attached to athlete 1’s ankle, knee, and coxa; (**c**) the output triboelectric voltage of MS-TENG attached to left/right coxa when athlete simulates curve-skating at different inclination angles; (**d**) the output triboelectric voltage of MS-TENG attached to ankle when athlete 1 simulates straight-skating; (**e**) athlete 1—detailed drawings of voltage curves simulating straight-skating; (**f**) the output triboelectric voltage of MS-TENG attached to ankle when athlete 2 simulates straight-skating; (**g**) athlete 2—detailed drawings of voltage curves simulating straight-skating.

On the basis of the superior performance of electrical output and splendid sensing property to force, MS-TENG can be used to monitor skaters’ motion techniques. The human movement system consists of bones, joints, and muscles. By using the flexibility of MS-TENG, it can be attached to the joints of the skater flexibly. With flexion and extension of the targeted joints, MS-TENG would then be compressed and released, converting mechanical signals into voltage signals simultaneously (Figure 4a). The oscilloscope synchronously collects the voltage signals of MS-TENG which is attached to the ankle, knee, and coxa of athlete 1 (as shown in Figure 4b). All the above sensors are 8 × 3 cm^2^ in size. The detailed collection process is shown in Movies S1–S3. In addition, athlete 2 also performed the same motion test, and the voltage signal is as shown in Figure S7. The results are summarized in Table 1. At the same joint motion, the output voltage of athlete 1 is higher, but his variance is large. To sum up, it shows that athlete 1 is a strength-type player, and his technical stability needs to be improved. Athlete 2 is a technique-type player, and his strength needs to be enhanced. Speed skating is a periodic event, and it is a fitness and technique sport. Speed skaters possess great physical strength and excellent technique. According to the monitoring results, the coach can arrange technical training for athlete 1 appropriately, so that athlete 1 can form the correct motion concept and maintain the good stability, thus he can improve his competitive ability. The coach can arrange strength training load for athlete 2 to ensure that he can adapt to the load requirements of the competition. To avoid being thrown off the track, the athlete can adjust his barycenter at curve-skating and push off the ice with the outside edge of his left skate and the inside edge of his right skate, which can keep his body tilted toward the center of the circle. In this state, the athlete uses the centripetal force that is formed by the supporting reaction force of the body’s barycenter and the resultant force of body gravity to counter the centrifugal force which is generated by circular motion. Figure 4c is the output voltage of MS-TENG which is attached to left/right coxa when athlete simulates curve-skating at different inclination angles. The incline angle formed by the body, and the ice is closely related to the athlete’s speed at curve-skating. The lower the incline angle, the smaller the skating radius, and the faster the speed. At high, moderate, and low inclination angles, the voltage of left coxa is 1.16, 1.54, and 1.78 V, and voltage of the right coxa is 1.01, 1.29, and 2 V. The result shows that the lower the incline angle, the higher the voltage. Figure 4d,f shows the output voltage of MS-TENG attached to ankle when two athletes simulate straight-skating. Detailed drawings of voltage curves are shown in Figure 4e,g. It shows that two athletes do leg extension-ankle extension-leg retraction movements. Because the sole of the Clap skate has a special hinge device (Figure S8), the heel of the skate can be separated from the skate. Therefore, athletes can take an action to extend their ankles in the process of pushing off the ice. This allows the edge to stay in contact with the ice longer; thus, it is important to improve the athlete pushing-off effect. As shown in Figure 4g, three signal waves correspond to the three movements of athlete 2’s leg-extension–ankle-extension–leg-retraction movements. However, only two signal waves of athlete 1’s leg-extension–leg-retraction can be observed in Figure 4e, and the above results show that the technical action of athlete 1 needs to be improved. With the rapid progress of science, the mobile phone has become a necessary tool in people’s lives. People collect a lot of information through mobile phones. If mobile phones can collect the motion monitoring information, it would be more convenient to monitor the motion. Therefore, a wireless sensor system consisting of a flexible MS-TENG, a digital multimeter with a Bluetooth module and a mobile phone was established to verify the feasibility of human motion monitoring (Movie S4). Through digital multimeter transmitting the flexible-sensor-collected signals, an app in the mobile phone can monitor the voltage change in real time. Through analysis of these data, the technical information of athletes can be learned, which could provide quantifiable, objective, accurate, and reliable support in sports training.

**Figure 5:**
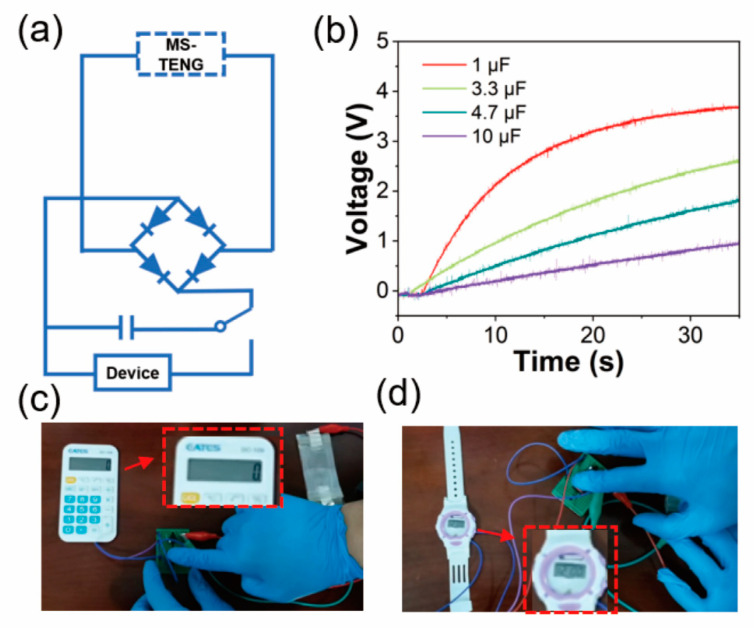
(**a**) The equivalent circuit of a self-powered system; (**b**) charging voltage of different capacitor which is charged by MS-TENG; (**c**) powering for an electronic calculator; (**d**) powering for an electronic watch.

The mechanical energy which is generated by human motion belongs to good-quality renewable forms of energy, because it is not limited by time, place, or other objective factors. meanwhile it is sustainable and easily accessible. The MS-TENG can be used to harvest biomechanical energy which is generated by human motions. Figure 5a shows the equivalent circuit of the self-charge system. The electrical energy output is from MS-TENG which can be stored in an energy storage device (such as a capacitor) to power electronic devices. Figure 3c shows the voltage–time curve that MS-TNEG charges different capacitors. It can charge 1, 3.3, 4.7, and 10 μF capacitors to 3.7, 2.6, 1.8, and 1 V when the frequency is 5 Hz for 35 s. MS-TENG charging a 4.7 μF capacitor is shown in Movie S5. As shown in Figure 5c,d, an electronic calculator and an electronic watch can work about 15 s after hitting the sensor (Movies S6 and S7). These demonstrations indicate that the MS-TENG has great potential in a fully self-powered and sustainable electronic system.

## 4. Conclusions

In summary, a flexible TENG based on a micro-structure (MS-TENG) is fabricated with a facile and low-cost fabrication method. Moreover, the fabrication method can be used as a universal strategy for improving the output of TENG. Through the micro-structure, the voltage of the MS-TENG can be im-proved by 7 times. We prepared a hydrogel to replace a traditional electrode to overcome the vulnerability of traditional metallic electrode in TENG during long-term service with large deformation. The MS-TENG provides outstanding sensing properties: maximum output voltage of 74 V, angular sensitivity of 1.016 V/degree, high signal-to-noise ratio and excellent long-term service stability. We used it to monitor the running skills of speed skaters. Based on the triboelectric effect, it can accurately convert the technical action information (such as motion, bending angle, and frequency) of athletes in training into triboelectric signals for outputting. Moreover, there is no external power supply for the whole process. In addition, MS-TENG can collect energy from human mechanical motion to drive small electronic devices (such as electronic calculators and electronic watches). The self-powered sensing and sustainable energy conversion realized by MS-TENG show its potential as a new generation of motion-monitoring equipment.

## Figures and Tables

**Table 1:** Comparison of athletes’ data.

	Athlete 1	Athlete 2
Average voltage of ankle	3.28 V	1.78 V
Variance of ankle voltage	4.817	0.203
Average voltage of knee	4.17 V	0.62 V
Variance of knee voltage	0.555	0.0135
Average voltage of coxa	0.9 V	0.45 V
Variance of coxa voltage	0.168	0.002

## Data Availability

The data presented in this study are available in Supplementary Materials.

## Supplementary Materials

The following supporting information can be downloaded at: https://www.mdpi.com/article/10.3390/nano12091576/s1, Figure S1: Scanning electron microscope (SEM) images of the PDMS (a), FEP (b), and hydrogel (c), respectively; Figure S2: Fourier-transform infrared (FTIR) spectrum of the PDMS, FEP, and hydrogel; Figure S3: The tensile strength measurement process of hydrogel; Figure S4: The electrical conductivity of hydrogel; Figure S5: Output current of the MS-TENG at a load resistance of 9 MΩ; Figure S6: The linear relationship of angles and voltages; Figure S7: The output triboelectric voltage of MS-TENG attached to athlete 2’s ankle, knee, and coxa; Figure S8: The Clap skate with a hinge device; Table S1: The self-powered flexible sensor comparison with other works; Movie S1: The output signal of the ankle is collected; Movie S2: The output signal of the knee is collected; Movie S3: The output signal of the coxa is collected; Movie S4: The wireless monitoring system consisting of MS-TENG, Bluetooth multimeter, and mobile phone; Movie S5: MS-TENG charges a 4.7 μF capacitor; Movie S6: Powering for an electronic calculator; Movie S7: Powering for an electronic watch.
