# Supplementary material for: A Flexible TENG Based on Micro-Structure Film for Speed Skating Techniques Monitoring and Biomechanical Energy Harvesting
Source: Nanomaterials (Basel). 2022 May 6;12(9):1576. doi: 10.3390/nano12091576 (PMC9103164; doi:10.3390/nano12091576)
Supplement: Supplementary file 1 [file nanomaterials-12-01576-s001.zip › nanomaterials-1663314-supplementary.pdf]

nanomaterials

MDPI

*Supplementary Materials*

# A Flexible TENG Based on Micro-Structure Film for Speed Skating Techniques Monitoring and Biomechanical Energy Harvesting

**Zhuo Lu [1], Changjun Jia [2,*], Xu Yang [3], Yongsheng Zhu [2], Fengxin Sun [2], Tianming Zhao [4], Shouwei Zhang [1,*] and Yupeng Mao [1,2,*]**

[1] School of Physical Education, Northeast Normal University, Changchun 130024, China; luz560@nenu.edu.cn
[2] Physical Education Department, Northeastern University, Shenyang 110819, China; 2001276@stu.neu.edu.cn (Y.Z.); 2171435@stu.neu.edu.cn (F.S.)
[3] Changchun Polytechnic Tourism School, Changchun 130022, China; yangxu0316@126.com
[4] College of Sciences, Northeastern University, Shenyang 110819, China; zhaotm@stumail.neu.edu.cn
* Correspondence: 2071367@stu.neu.edu.cn (C.J.); zhangsw178@nenu.edu.cn (S.Z.); maoyupeng@pe.neu.edu.cn (Y.M.)

**Citation:** Lu, Z.; Jia, C.; Yang, X.; Zhu, Y.; Sun, F.; Zhao, T.; Zhang, S.; Mao, Y. A Flexible TENG Based on Micro-Structure Film for Speed Skating Techniques Monitoring and Biomechanical Energy Harvesting. *Nanomaterials* **2022**, *12*, 1576. https://doi.org/10.3390/nano12091576

Academic Editor: Christian M. Julien

Received: 17 March 2022
Accepted: 05 May 2022
Published: 6 May 2022

**Publisher's Note:** MDPI stays neutral with regard to jurisdictional claims in published maps and institutional affiliations.

**Copyright:** © 2022 by the authors. Licensee MDPI, Basel, Switzerland. This article is an open access article distributed under the terms and conditions of the Creative Commons Attribution (CC BY) license (https://creativecommons.org/licenses/by/4.0/).

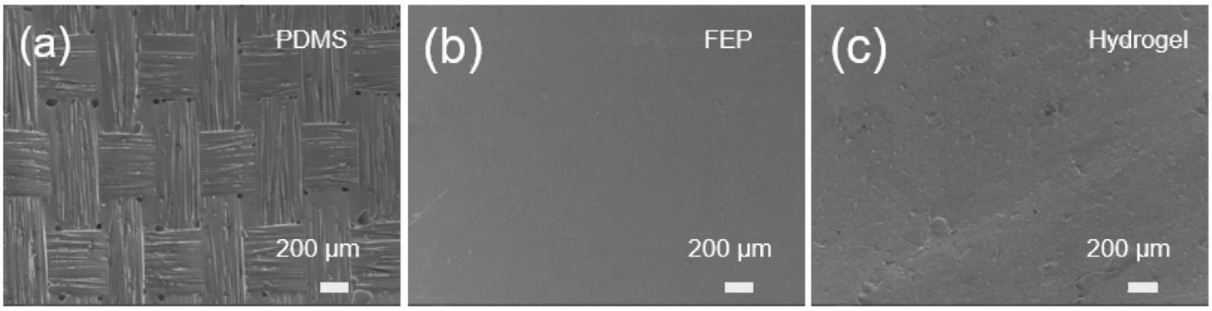


**Figure S1.** Scanning electron microscope (SEM) images of the PDMS (**a**), FEP (**b**), and hydrogel (**c**), respectively.

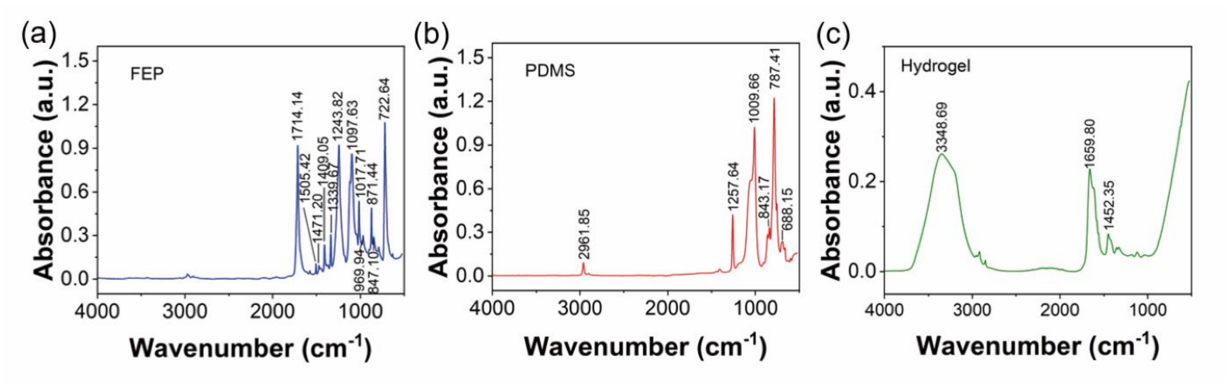


**Figure S2.** (**a**–**c**) Fourier-transform infrared (FTIR) spectrum of the PDMS, FEP, and hydrogel.

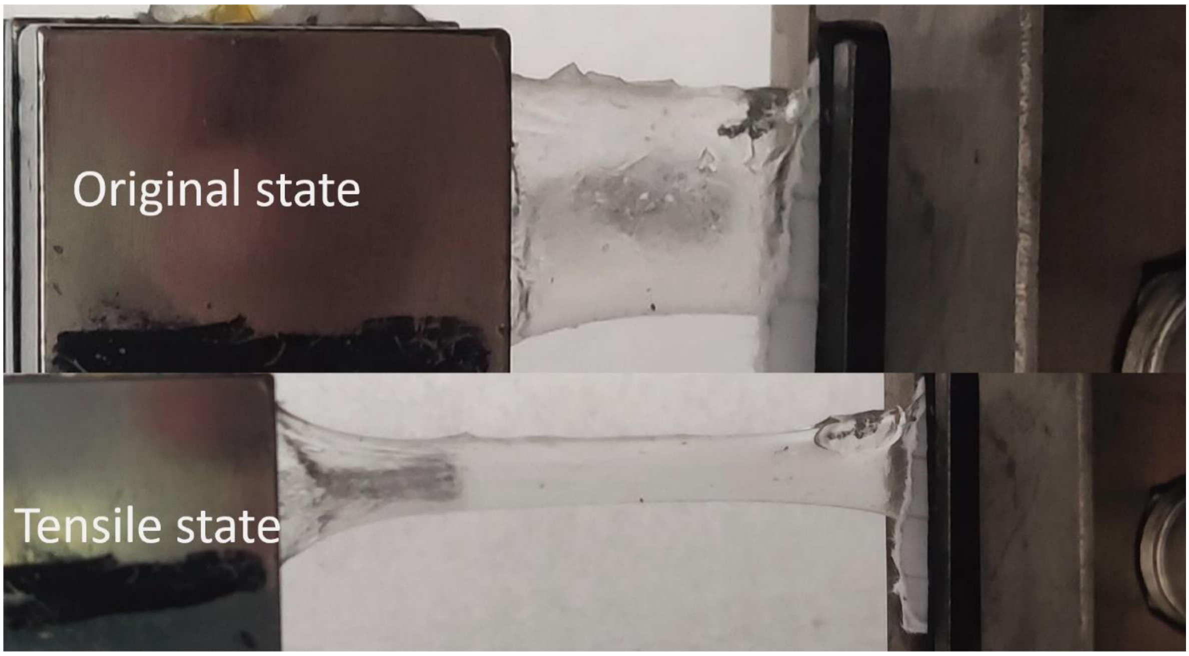


**Figure S3.** The tensile strength measurement process of hydrogel.

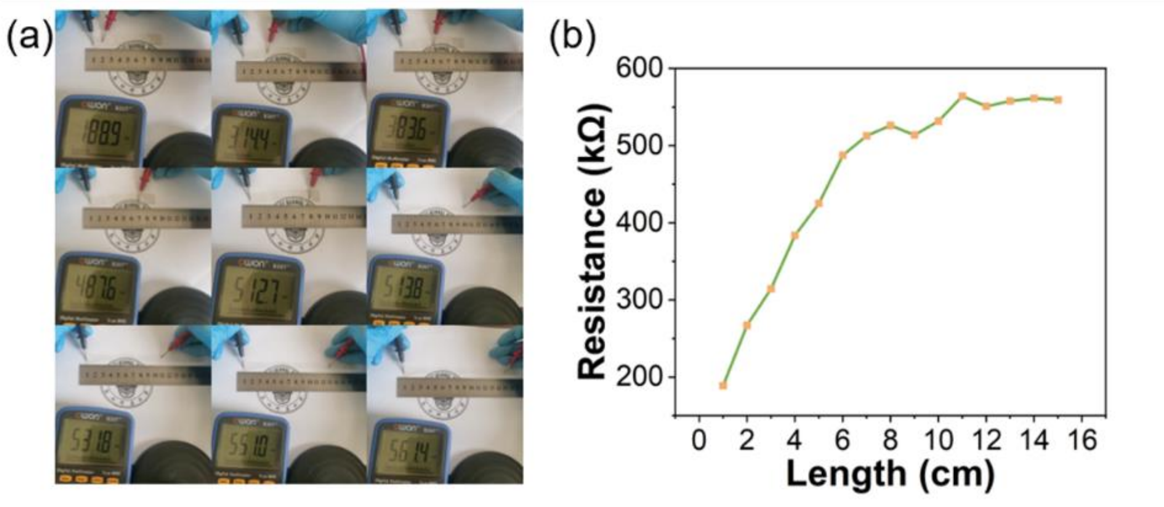


**Figure S4.** The electrical conductivity of hydrogel. (**a**) Measurement process of resistance; (**b**) resistance of hydrogels at different lengths.

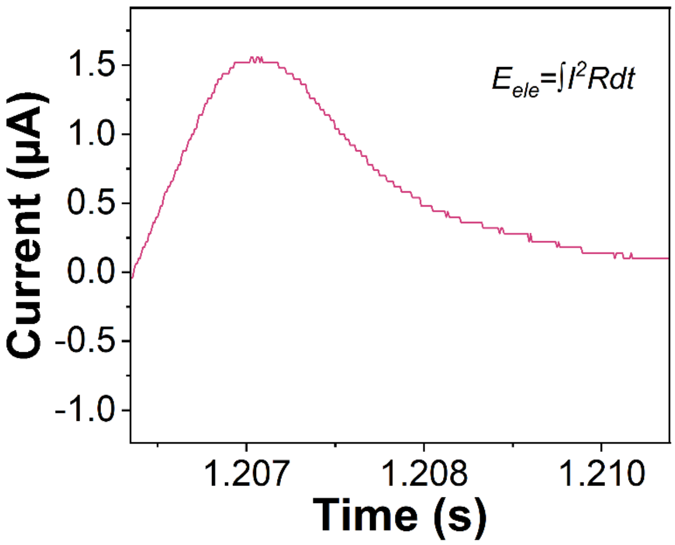


**Figure S5.** Output current of the MS-TENG at a load resistance of 9 MΩ.

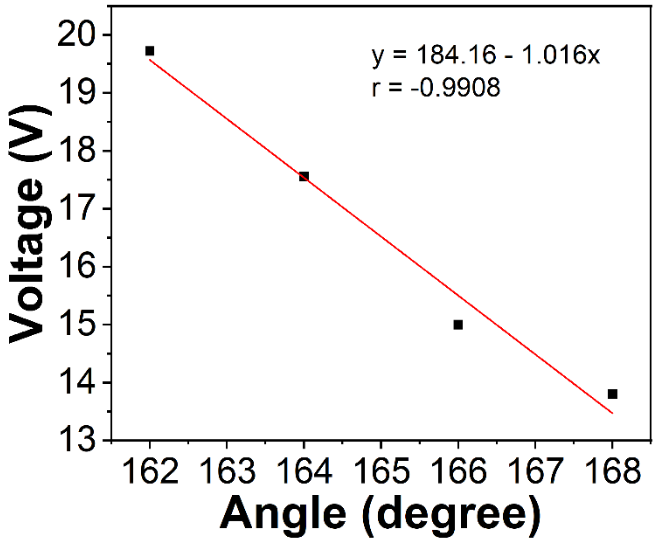


**Figure S6.** The linear relationship of angles and voltages.

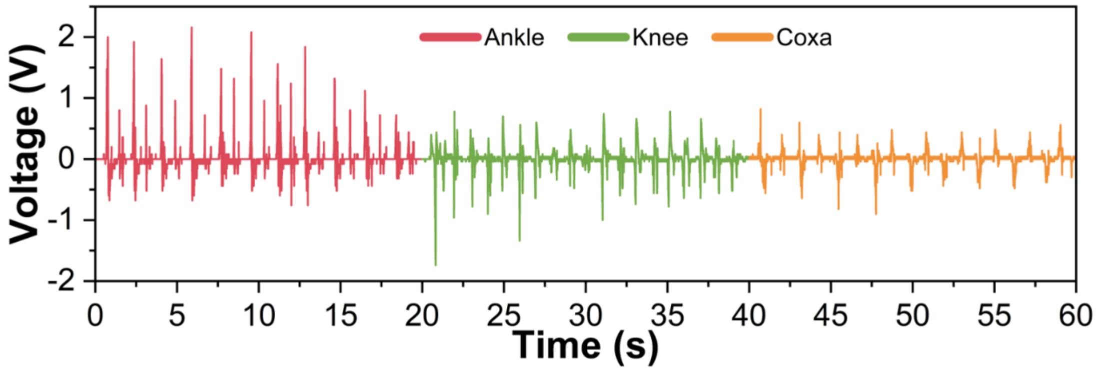


**Figure S7.** The output triboelectric voltage of MS-TENG attached to athlete 2's ankle, knee, and coxa.

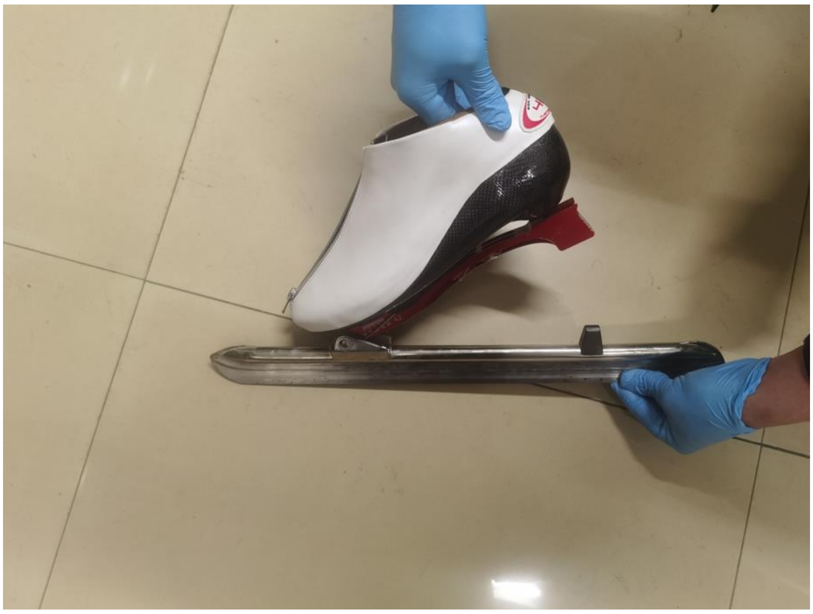

**Figure S8.** The Clap skate with a hinge device.

**Table S1.** The self-powered flexible sensor compared with other works.

| | **Our work** | **Other 1** | **Other 2** | **Other 3** | **Other 4** | **Other 5** | **Other 6** | **Other 7** | **Other 8** |
|---|---|---|---|---|---|---|---|---|---|
| Power supply mode | Triboelectric | Piezoelectric | Battery supply | Triboelectric | Triboelectric | Piezoresistive | Piezoelectric | Triboelectric | Triboelectric |
| Soft | Yes | Yes | No | Yes | Yes | Yes | Yes | Yes | Yes |
| Maximum output voltage | 74 V | 12 V | No | 50 V | 64 V | No | 11 V | 50 V | 250 V |
| Energy harvesting | Yes | No | No | Yes | Yes | No | No | Yes | Yes |
| Electrode | Hydrogel | Metal | Metal | Metal | Metal | Graphene Inks | Metal | Metal | Hydrogel |
| Doping modification | No | Yes | Yes | No | No | Yes | Yes | Yes | No |
| Device structure | 200 μm | 1 μm | 10 μm | 100 μm | 100 μm | 200 μm | 200 μm | 50 μm | 2 cm |
| Monitoring range | Joint activity | Heart activity, voice | Respiration, heart activity, walking, jumping, body temperature | No application | Gait | Joint activity | Finger bending | Joint activity | Joint activity |
| Reference | | 37 | 38 | 39 | 40 | 41 | 42 | 43 | 44 |

**Supplementary Movie S1.** The output signal of the ankle is collected;

**Supplementary Movie S2.** The output signal of the knee is collected;

**Supplementary Movie S3.** The output signal of the coxa is collected;

**Supplementary Movie S4.** The wireless monitoring system consisting of MS-TENG, Bluetooth multimeter, and mobile phone;

**Supplementary Movie S5.** MS-TENG charges a 4.7 μF capacitor;

**Supplementary Movie S6.** Powering for an electronic calculator;

**Supplementary Movie S7.** Powering for an electronic watch.
